# Current and future strategies against cutaneous parasites

**DOI:** 10.1007/s11095-022-03232-y

**Published:** 2022-03-21

**Authors:** Ernest Man, Helen P. Price, Clare Hoskins

**Affiliations:** 1 Pure and Applied Chemistry, University of Strathclyde, 99 George Street, Glasgow, G1 1RD UK; 2 School of Life Sciences, Keele University, Huxley Building, Keele, ST5 5BG UK

**Keywords:** anti-parasitic strategies, biomaterials, cutaneous, nanotechnology, parasites

## Abstract

Cutaneous parasites are identified by their specific cutaneous symptoms which are elicited based on the parasite’s interactions with the host. Standard anti-parasitic treatments primarily focus on the use of specific drugs to disrupt the regular function of the target parasite. In cases where secondary infections are induced by the parasite itself, antibiotics may also be used in tandem with the primary treatment to deal with the infection. Whilst drug-based treatments are highly effective, the development of resistance by bacteria and parasites, is increasingly prevalent in the modern day, thus requiring the development of non-drug based anti-parasitic strategies. Cutaneous parasites vary significantly in terms of the non-systemic methods that are required to deal with them. The main factors that need to be considered are the specifically elicited cutaneous symptoms and the relative cutaneous depth in which the parasites typically reside in. Due to the various differences in their migratory nature, certain cutaneous strategies are only viable for specific parasites, which then leads to the idea of developing an all-encompassing anti-parasitic strategy that works specifically against cutaneous parasites. The main benefit of this would be the overall time saved in regards to the period that is needed for accurate diagnosis of parasite, coupled with the prescription and application of the appropriate treatment based on the diagnosis. This review will assess the currently identified cutaneous parasites, detailing their life cycles which will allow for the identification of certain areas that could be exploited for the facilitation of cutaneous anti-parasitic treatment.

## Introduction

Parasites are organisms that are dependent on their host for survival and physiological development in a malignant manner and can be classified into three specific types, helminths, ectoparasites and protozoa. Helminths are large multicellular organisms that fall within 3 subcategories, platyhelminths, acanthocephalans and nematodes. Ectoparasites are a broad classification which encompasses arthropods such as mosquitoes, but typically refers to smaller arthropods such as mites, ticks and fleas. Lastly there are protozoa which are unicellular organisms that can multiply within their human host. Protozoa can be classified into four types with respect to their migratory action: Sarcodina, mastigophoran, ciliophoran and sporozoan.

The presence of the parasite increases the risk and spread of injury within the infected area, thus leading to the possibility of host mortality in the most severe of cases. The introduction of parasites into the host body can occur via a wide range of different routes such as the ingestion of contaminated food and water, transmission via an arthropod vector, intercourse, open wounds, an infected organ transplant etc. For the purposes of this review, only parasites that are transmitted via open wounds or vectors will be reviewed as these follow the route of infection via the transcutaneous pathway. Here we will discuss current treatment options and to serve as a call to action to the pharmaceutical community to translate medical technologies currently used to treat other diseases into pragmatic and cost effective treatments in the combat against cutaneous parasite infection.

## Cutaneous parasites

The vast majority of cutaneous parasites are transmitted via the direct skin contact of the human host with an infected vector or is facilitated by contact with a piece of contaminated material containing the parasite. The infected medium is normally a vector or a form of contaminated material, such as soil or clothing infested with the parasitic eggs. Specific types of parasites are capable of laying eggs, i.e., helminths and ectoparasites. In most instances the parasitic eggs are laid within the epidermal layer of the human skin, where they begin hatch and mature, resulting in escalated cutaneous damage. Initial symptoms may include irritation and inflammation as the parasitic eggs are ejected into the skin. Once the eggs hatch, the larval parasites may move around the cutaneous and subcutaneous layers, resulting in lesions and minor haemorrhaging beneath the skin. There are also other instances where parasitic eggs are introduced into the human host through non-cutaneous means but can instead occur through the ingestion of contaminated sustenance, which then develops and migrates within the host leading to cutaneous symptoms, i.e., gnathostomiasis. Of the three classifications, protozoans are the only ones that do not lay eggs, but instead multiply within the host, which when coupled with their specific migratory mode, can result in observable cutaneous skin symptoms. Such an example would leishmaniasis. It should be noted that not all parasites that are transmitted via skin contact will result in cutaneous pathologies, however for the purposes of this review we will specifically be exploring the parasites that elicit cutaneous symptoms, Table I.

**Table I:** List of cutaneous parasites and their transmission methods.

Parasitic infestation	Associated parasite	Transmission method	Reservoir	References
Gnathostomiasis	*Gnathostoma* genus	-Ingestion of infected aquatic animal	-Fresh water copepods -Certain fish species -Certain aquatic animals	(1, 2)
Leishmaniasis	*Leishmania amazonensis, Leishmania braziliensis,* *Leishmania guyanensis, Leishmania major*, *Leishmania Mexicana, Leishmania panamensis,* *Leishmania tropica, Leishmania aethiopica*	-Bite from an infected female sand-fly from the *Phlebotomus* species	-Mammals	(3, 4)
Lyme disease	*Borrelia burgdorferi*, *Borrelia afzelii*, *Borrelia garinii*	-Bite from a tick of the *Ixodes* genus	-Human	(5, 6)
Myiasis	*Dermatobia hominis*, *Cordylobia anthropophaga*, *Cuterebra spp, Cochliomyia hominivorax,* *Chrysomya bezziana, Wohlfahrtia magnifica,* *Gasterophilus spp, Hypoderma ovis, Hypoderma lineatum*	-Skin contact with soil or clothing infested with parasitic eggs	-Human	(7, 8)
Onchocerciasis	*-Onchocerca volvulus*	-Bite from an infected black fly of the *simulium* genus	-Human	(9, 10)
Pediculosis	*Pediculus humanus var. capitis, Pediculus humanus var. pubis*	-Direct and indirect skin to skin contact	-Human	(11, 12)
Scabies	*Sarcoptes scabiei*	-Direct and indirect skin to skin contact	-Human	(13, 14)
Strongyloidiasis	*Strongyloides stercoralis, Strongyloides fuelleborni*, *Strongyloides myopotami, Strongyloides procyonis*	-Skin contamination by soil infected with the filariform larvae	-Human	(15, 16)
Tungiasis	*Tunga penetrans*, T*unga trimamillata*	-Cutaneous ectopic penetration by the female sand flee	-Human -Domestic animals -Sylvatic animals	(17, 18)

### Leishmaniasis

Leishmaniasis is caused by the bite and subsequent ejection of the Leishmania promastigotes into the human host by the female *Phlebotomus* sandfly. The leishmania disease itself is based off of *Leishmania spp*, which are unicellular eukaryotes called protozoans that multiply within the host.

The initial bite induces an inflammatory response at the site of injury, recruiting professional phagocytes to the location, which then subsequently phagocytose the foreign promastigotes. Typically speaking once a phagocytes compartmentalises foreign matter, they fuse with the lysosomes resulting in the release of reactive oxygen species (ROS) which destroys the foreign material. In the case of *leishmaniasis*, the parasite utilises neutrophils as a medium to safely facilitate its undetectable entry into host macrophages (19). The utilisation of neutrophils by leishmania occurs via the protozoan interfering with the granule fusion process within the neutrophil, which in turn prevents the neutrophil from generating microbicidal granules which would kill the protozoan (20) This was observed in the first day of *L.mexicana* and *L.Major* infection, whereby the protozoan were observed to be within the host neutrophils, evidencing the resistance that leishmania had developed against the neutrophil’s microbicidal processes (21). Once the leishmania promastigotes have been transported into the phagocyte, they then begin to inhibit the fusion of the host phagocyte with the lysosome, via the process of phagosome maturation arrest. This then results in an isolated space devoid of ROS, which allows the promastigote to replicate within the phagocyte, thus becoming an amastigote (22).

**Fig. 1:**
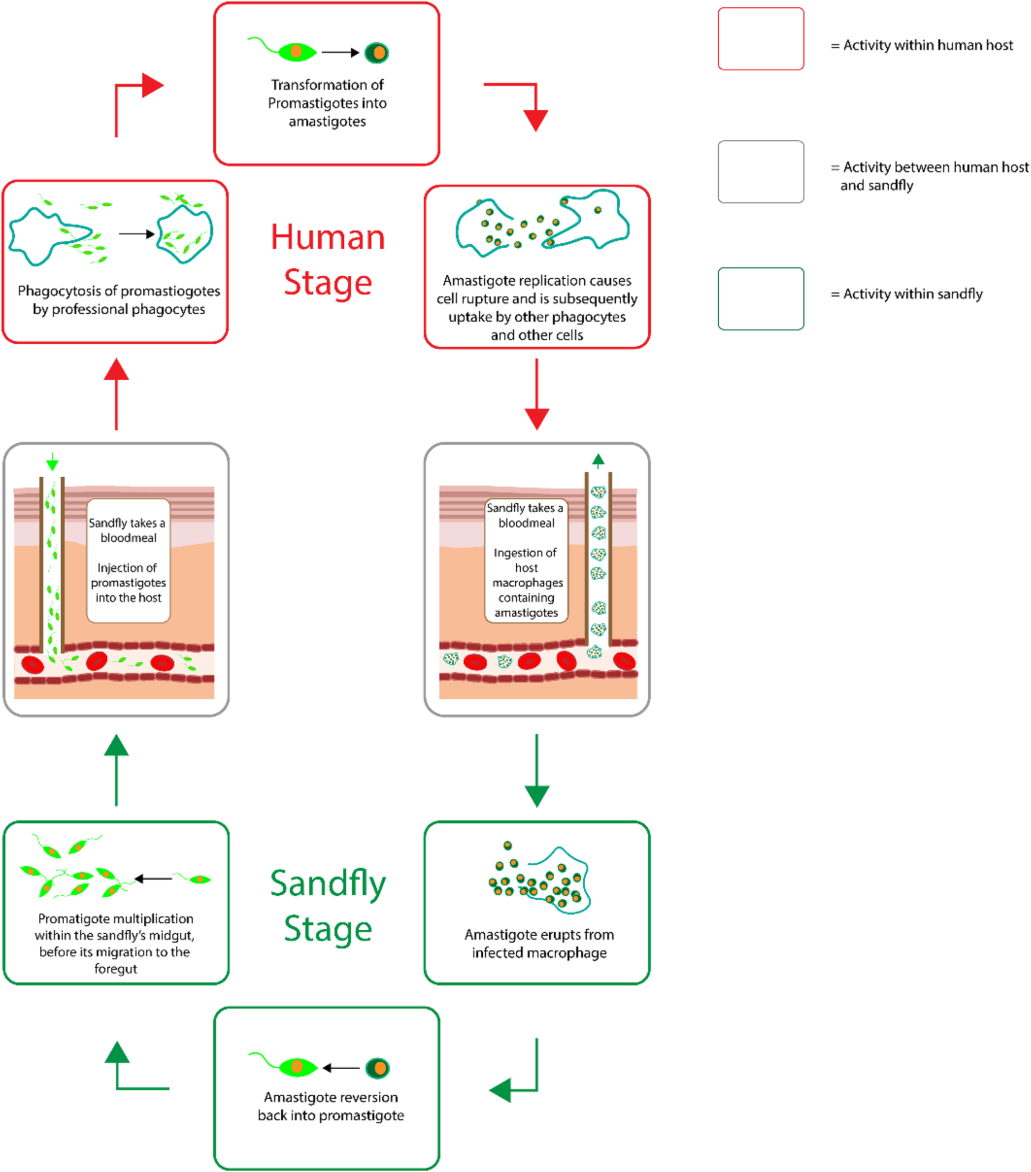
The life cycle of *Leishmania*.

Eventually the amastigotes replicate up to a certain point in which their numbers begin to affect the physiological stability of the phagocyte leading to cellular rupture, accompanied by the subsequent uptake of amastigotes by uninfected phagocytes. The amastigotes then begin to circulate throughout the host, eliciting responses with respect to the type of leishmania species within the host. In the specific case of cutaneous leishmaniasis, the species of *Leishmania amazonensis, Leishmania braziliensis, Leishmania guyanensis, Leishmania major, Leishmania mexicana, Leishmania panamensis,* and *Leishmania tropica* result in cutaneous responses which then escalate in accordance with the magnitude of division undertaken by the parasite. The circulation of the amastigotes within its host only represents one part of its life cycle as the *Phlebotomus* sandfly consumes the blood of its host, thus intaking the parasitized phagocytes within itself, which initiates the second part of the *Leishmania* life cycle. Once the amastigotes are taken up by the sand-fly, they differentiate back into promastigotes which then replicate and migrate through midgut and foregut of the sand-fly to the salivary glands, allowing the promastigotes to be transmitted into the next host, thus repeating the lifecycle, Fig. 1.

The initial bite caused by the sand-fly instigates an acute inflammatory response which quickly subsides, however there are typically no noticeable symptoms post-skin trauma for several weeks and it is this which represents the promastigote incubation phase. Prior to the manifestation of cutaneous symptoms, lymphadenopathy may occur, which can act as an early indicator of human *leishmaniasis.*

The cutaneous symptoms presented by this disease occur as a result of amastigote metastasization which triggers leukocytic and fibroblastic activity in response to the detection of infected phagocytes. The activation of leukocytes and fibroblasts result in localised damage to the structural architecture of the native tissue, causing keratinocyte necrosis and apoptosis, which in turn leads to the occurrence of dermal tissue necrosis and therefore the visual manifestation of dermal injury. The initial dermal symptoms typically manifest as a lesion at the initial site of the sandfly bite, which occurs most commonly on the more exposed regions of the human body, including the lower legs, arms, neck and face. As the disease escalates, the lesion may progress into nodules, ulcers and various plaque formations, thus representing the ulcerative phase of disease.

These cutaneous lesions often self-heal over time, however specific species of *Leishmania* can produce clinical symptoms of differing severity and recovery periods. I.e. *L.major* more than 50% of patients self-heal in 2–8 months, *L.tropica* self-heals after 1 year, *L.aethiopica*, self-heals within 2–5 years, *L.mexicana* often self-heals within 3–4 months and *L.Braziliensis* self-heals between several months to years (http://www.emro.who.int/neglected-tropical-diseases/information-resources-leishmaniasis/cl-factsheet.html) (23, 24). In the case of *Leishmania aethiopica, Leishmania amazonensis, Leishmania braziliensis* and *Leishmania panamensis,* the escalation of leishmaniasis can result in the transition of cutaneous manifestations into mucocutaneous symptoms which can result damage nasal and oral tissue, with disruptions to regular respiratory function occurring in more severe cases. Another instance of a *Leishmania* species producing irregular cutaneous symptoms is *Leishmania amazonensis,* which produces simultaneous nodular lesions in multiple locations of differing sizes and is often identified in areas of the body away from the primary lesion caused by the bite of sand-fly.

Cutaneous *leishmaniasis* can be represented by 7 distinct pathophysiological phases, starting with the acute phase caused by the initial bite by the infected sand-fly followed by the silent phase where the promastigotes begin to replicate slowly. The next phase is the active phase which involves the initial development of necrotic tissue due to the increased recruitment of phagocytes. This eventually escalates to the ulcerative phase which is characterised by mass tissue necrosis. Once necrosis begins to slow down, the healing phase initiates, resulting in the re-epithelialization of the damaged dermal structures, however this may result in ‘over-healing’ leading to the occurrence of hyperplasia which represents the chronic phase. Ultimately this develops into the final phase, non-ulcerative leishmaniasis which is represented by the minor atrophy of the epidermal layer (25).

### Lyme’s disease

Lyme’s disease, which is also known as *Lyme borreliosis* is the disease associated with the bite from an *Ixodes* tick infected with the bacteria *Borrelia*. Typically speaking, the transmission of this disease requires the tick to be attached onto the host for a minimum of 36 h and is separated into three distinct phases, starting with the early localized disease, which is characterised by the cutaneous development of erythema migrans at the bite area, coupled with systemic symptoms such as fever, malaise and headaches. The next phase is the early disseminated, where the singular erythema migran develops into multiple erythematic lesions with possible systemic symptoms such as lymphadenopathy, cranial nerve palsies and possible cardiac abnormalities. The final phase is the late disseminated, which does not involve the escalation of cutaneous abnormalities, but instead results in the development of arthritis in major joints. In terms of the cutaneous pathophysiological development of *Lyme borreliosis*, the erythema migrans typically appears after one to two weeks of the initial bite and can expand to a diameter size of + 5 cm over several days, with the possible development of visible concentric rings around the bite area. This generally persists up to 3 weeks if untreated, exuding a burning or itching sensation, but some cases may be asymptomatic. The main cutaneous hallmark of *Lyme borreliosis* is the development of concentric rings at the bite area, which is visually comparable to a bullseye. The *Borrelia* infection can usually be eliminated via a course of antibiotics lasting 2–4 weeks. However, if the infection is not treated early on, it can lead to neurological damage, with some rare cases of the symptoms persisting for up to 6 months.

**Fig. 2:**
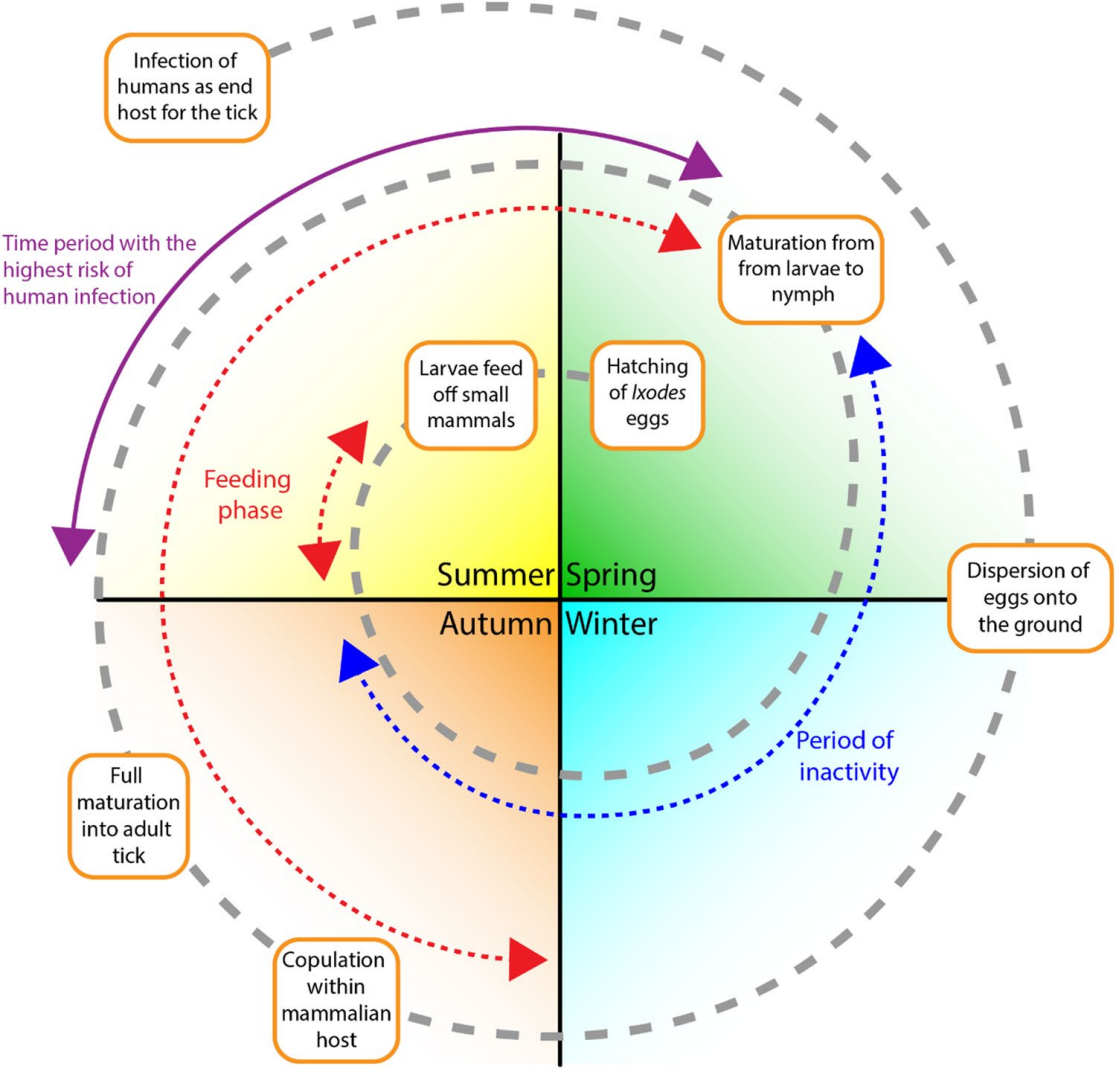
The life cycle of the Ixodes tick.

In regard to the development of *Lyme Borreliosis*, it is highly dependent on the location and life cycle of the *Ixodes* tick. The geographical distribution of the disease is biased towards the northern regions with temperate climates that contain a consistently high level of relative humidity. This is important as it affects the survival of the bacteria and the tick vector but can also determine the mammalian host in which it feeds and develops from. In most cases the tick itself feeds and copulates on deer and mice during autumn or spring, whereby the female tick then detaches from the mammalian host, laying its eggs on the ground which then hatch prior to summer. During the summer season, the newly hatched larvae feed on small mammals such as rodents and birds, before becoming inactive until the following spring. Upon the arrival of the next spring, these larvae then develop into nymphs which then repeat the same feeding cycle as their larval forms, before finally developing into adult ticks in autumn (https://wonder.cdc.gov/wonder/prevguid/p0000380/p0000380.asp#head001003000000000). Overall, the developmental cycle takes approximately 2 years prior to reach full maturation, however during this time the tick may infect domestic animals which can then be transferred onto a human host, thus resulting in human based Lyme’s disease, Fig. 2.

### Myiasis

Myiasis is the disease resulting from the infestation of the human host by certain species of fly. Different species of fly produce differing forms of cutaneous myiasis: *Cordylobia anthropophaga, Cuterebra spp., Dermatobia hominis,* and *Wohlfahrtia vigil* result in furuncular myiasis; whereas *Gasterophilus spp., Hypoderma ovis* and *Hypoderma lineatum* result in migratory myiasis; and finally *Chrysomya bezziana*, *Cochliomyia hominivorax* and *Wohlfahrtia magnifica* which result in traumatic myiasis. Each of these three types of cutaneous myiasis can be distinguished by their distinct visual characteristics, which can be used as a form of identification to determine the infective species in question. Furuncular myiasis is characterised by the presence of erythematic lesions in the form of single or multiple papules, which then develop in furuncles as larvae grow. Migratory myiasis in contrast is characterised by lesions that occur as a result of larval migration, thus resulting in the occurrence of an elongated wound that may take on a meander-like morphology. Finally, there is traumatic myiasis which is the most severe form of myiasis, which revolves around the parasitic larvae infecting pre-existing wounds, causing significant cutaneous and subcutaneous haemorrhaging to the host tissue. This specific form of myiasis can be life-threatening if the larvae burrow into an existing wound on the host’s facial area, as it can then further penetrate into ocular and brain tissue, resulting in blindness, possible brain damage, sepsis and death. The geographical distribution of human cutaneous myiasis is typically biased towards the tropical regions with more temperate climates, with the highest occurrence in regions of extreme poverty and low levels of hygiene (26).

**Fig. 3:**
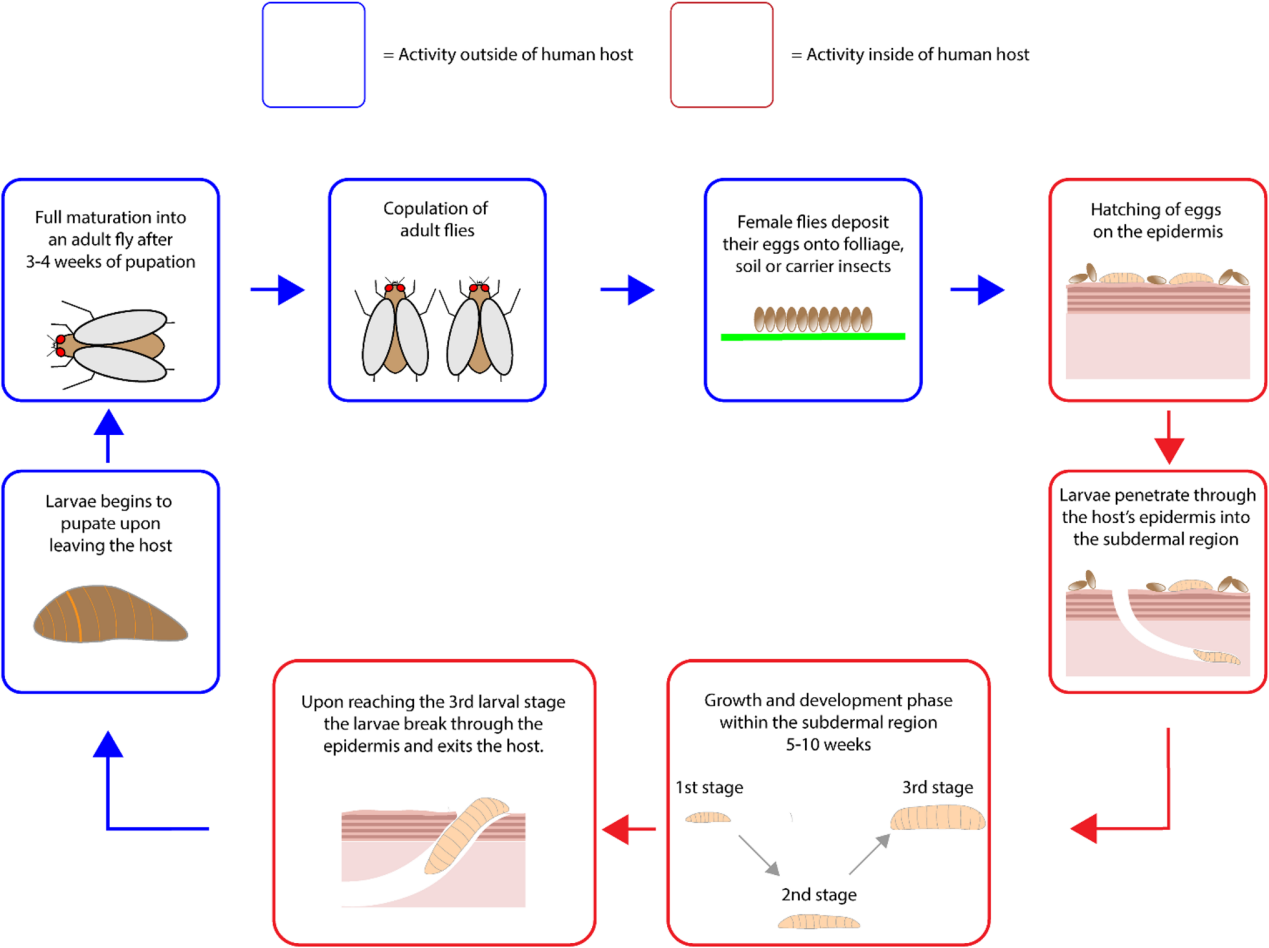
The life cycle of myiasis larvae.

In regards to the life cycle of myiasis causing parasites, it is specific to the species associated with a particular type of cutaneous myiasis. In the case of furuncular myiasis, the main vector is *Dermatobia hominis*, which deposits its eggs onto either foliage or carrier flies. Once these parasitic eggs reach the host epidermis, they hatch and penetrate into the subdermal region where they begin to grow and develop for 5–10 weeks (27). Upon maturation, the larva pierces through the epidermis where it then detaches itself from the host before pupation. *Cordylobia anthropophaga* is also another vector associated with furuncular myiasis, but unlike *D. hominis*, follows a different route of human infestation. In the case of *C. anthropophaga,* the eggs are deposited onto contaminated soil which then hatch into larvae, before burrowing into the ground for approximately 9 days. During this period, the larvae will detect the presence of a possible host based on ground vibrations and heat signatures before attaching themselves onto the host. Once the host has been parasitized, then begins to burrow into its host tissue for one and a half weeks, causing furuncular myiasis in the process. Eventually the parasite leaves its host via a lesion, where it then begins to pupate before hatching into an adult fly. In the case of migratory myiasis, the two most common parasites are *Gasterophilus* species, *Hypoderma ovis* and *Hypoderma lineatum* which both begin the route of infestation by having their eggs laid on domestic animals. For *Gasterophilus* species, the parasite first lays their eggs on horse hairs which then hatch into larvae that burrow into the epidermis of human hosts upon successful contact. The parasite itself burrows into the lower epidermis where it then begins to migrate at that level, causing raised lesions that represent the parasite migratory route. Unlike the *Gasterophilus* species, *Hypoderma ovis* and *Hypoderma lineatum* are both slightly more severe in terms of their migratory routes which penetrate deep into the subcutaneous tissue of the host, damaging a large variety of areas including the muscle tissue, skin and nerve fibres. Both *H. ovis* and *H. lineatum* begin by attaching their eggs onto cattle hair, which then proceed to hatch within a week. The resulting larvae then burrows into the subcutaneous region causing erythema lesions, before migrating to other areas of the host body. For traumatic myiasis the most commonly associated species are *Wohlfahrtia magnifica, Chrysomya bezziana* and *Cochliomyia hominivorax*. In the case of *W. magnifica*, the vector deposits larvae directly onto the pre-existing cutaneous lesion which then proceed to feed for up to a week, resulting in major injury to the surrounding tissue. After the feeding phase, the larvae detach from the host and begins to pupate. For *C. Bezziana* and *C. hominivorax*, the vector deposits its eggs on the periphery of the lesion which then hatch after 15 h of incubation, before entering the feeding phase (27). Although the feeding phase is known to cause significant tissue damage to the host, the main problem lies in the quantity of parasitic eggs deposited by the vector, ranging from 150–500 eggs which result in the occurrence of multiple infestation cases. The feeding phase persists for up to one week, before the larvae detach from the host to begin pupation, Fig. 3.

### Pediculosis

**Fig. 4:**
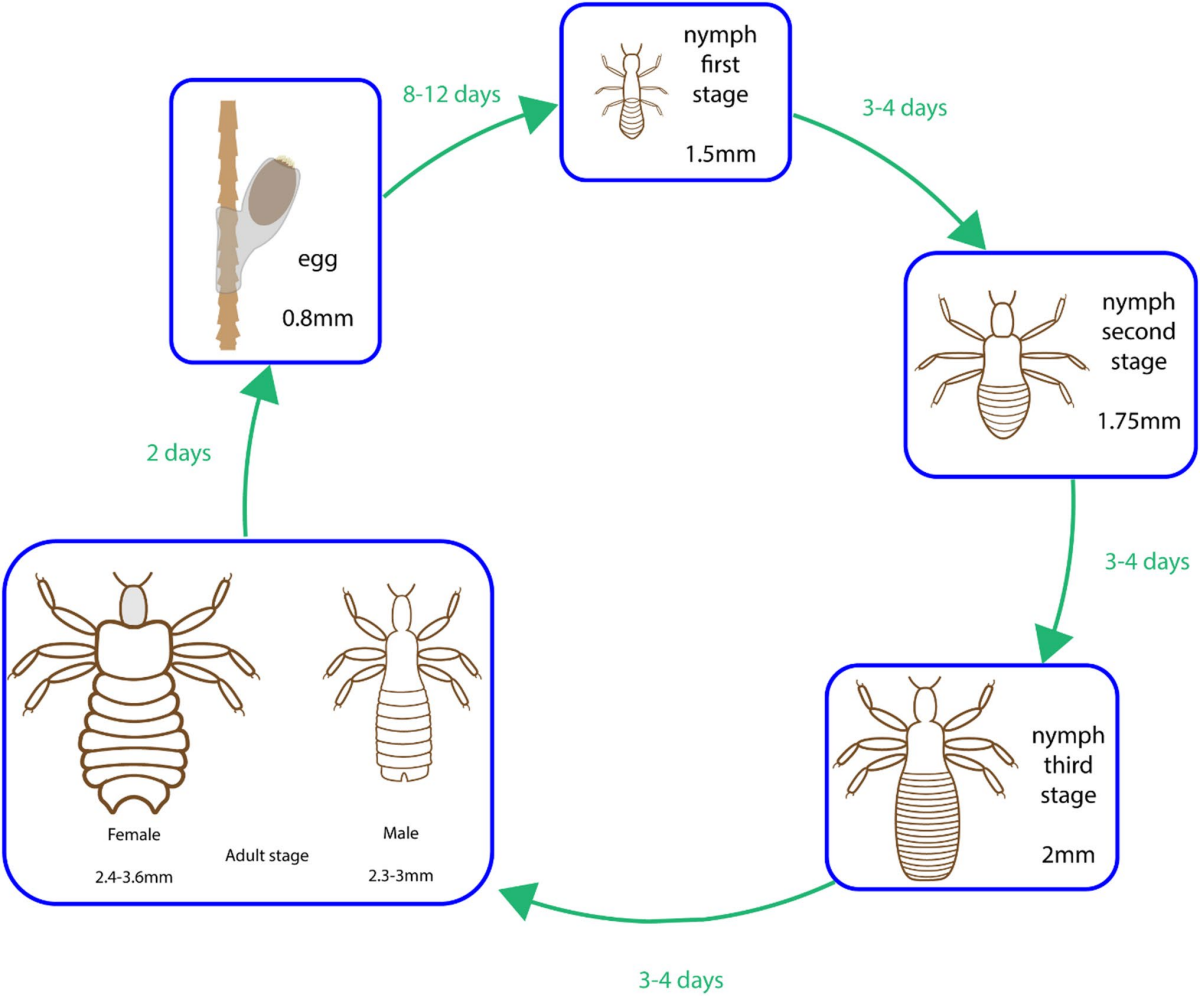
The life cycle of *Pediculus humanus var. capitis*.

Pediculosis is the cutaneous parasitic infestation of the human scalp by the louse *Pediculus humanus var. capitis*. The life cycle of this parasite can be separated into the three stages of egg, nymph and adult, in which the adult stage is responsible for infestations and the main clinical manifestation. The cycle begins with its human host being infected with an adult louse which can remain within the scalp region for up to 30 days, Fig. 4. During its infestation period, both adult male and female lice feed on their host’s blood which generally does not trigger any significant clinical manifestations, however some hosts may develop an allergic reaction to the saliva of the louse and can also develop secondary bacterial infections with respect to the pathogen carried by the parasite.

The life cycle continues with the adult female louse laying up to eight eggs daily onto a hair shaft of the host, which take roughly a week to hatch. Once hatched, the nymph goes through three stages of moulting before reaching its adult stage within the course of a week. The general mode of transmission is head-to-head contact of hosts, however secondary forms of transmission can also occur via the transfer of the louse onto items that are of close proximity to the head of the infected host, for example combs, pillows and towels. Regarding the global distribution of cases, pediculosis is widely distributed globally with significantly greater prevalence in children aged 3 to 11 (https://www.cdc.gov/dpdx/pediculosis/index.html).

### Scabies

Scabies is the cutaneous disease associated with the migration of the burrowing mite *Sarcoptes scabiei*, into the skin of the host organism. The primary transmission route is direct skin to skin contact between a host infected with fertile female mites and a new host. Secondary transmission routes can also occur via day-to-day fomites that are of close proximity to the epidermis, such as clothing, bedding and furniture. Scabies exists as a result of the immunological response to the presence of *Sarcoptes scabiei* within the epidermal layer of the host’s skin. The parasite itself only burrows within the epidermis, never going below the stratum corneum (https://www.cdc.gov/parasites/scabies/biology.html). The immunological response to the parasite typically manifests itself in the form of pruritus with visible signs of raised lines where the mites are present. The manifestation of such symptoms may not appear for up to two months from the initial infestation period. However, during this time, infestation is perpetuated by the life cycle of the parasite, leading to the possible accumulation of delayed cutaneous symptoms.

**Fig. 5:**
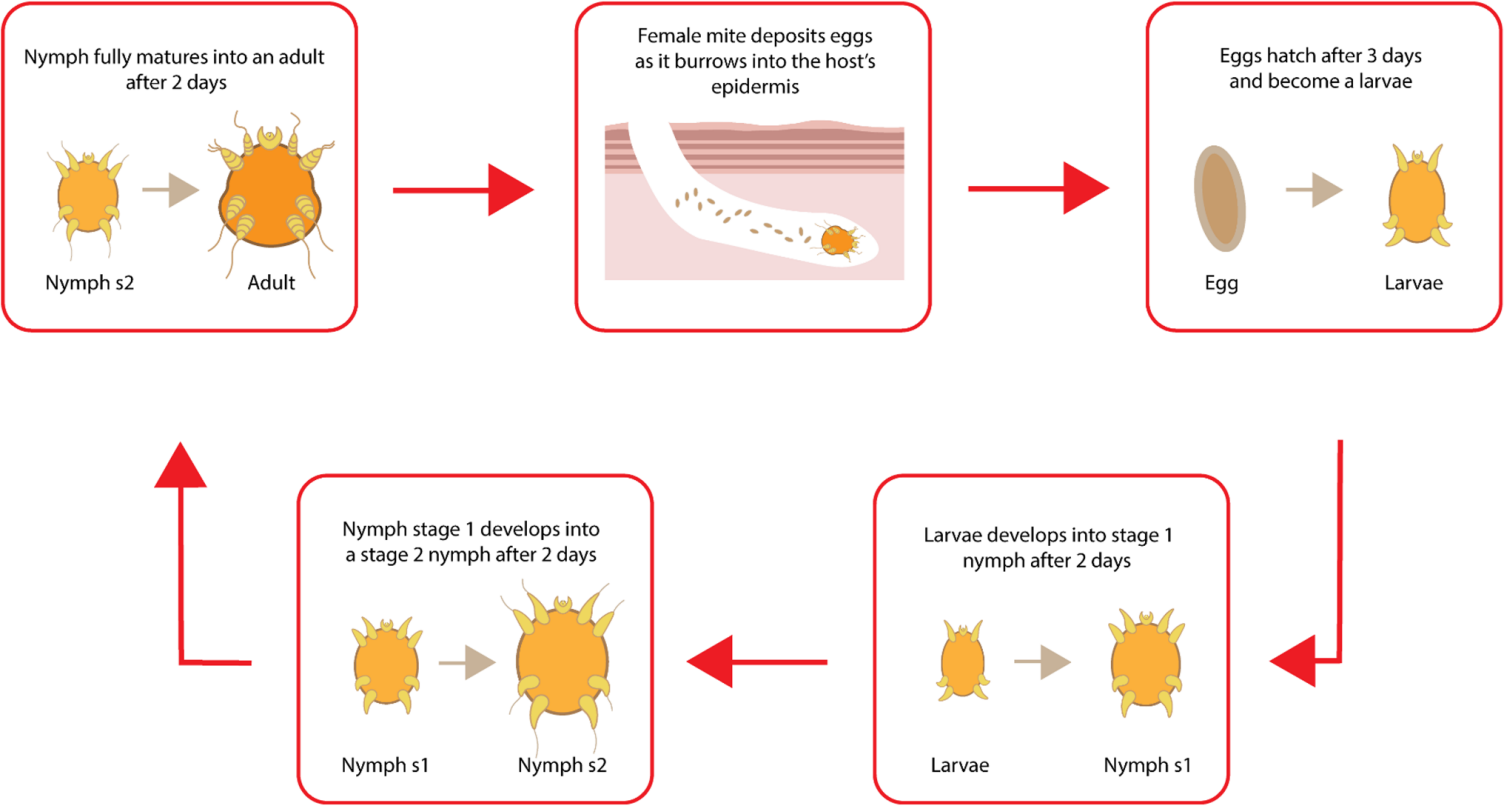
The life cycle of *Sarcoptes scabiei*.

Secondary immunological responses can also arise due to the presence of specific bacterial species carried by the parasite. The initial site of infestation can become infected by skin-based commensal bacteria such as *Staphylococcus epidermidis* and *Staphylococcus aureus* which may cause the initial wound site to degenerate into a chronic wound. The life cycle of *Sarcoptes scabiei* begins with the egg stage, whereby the eggs laid within the wound site begin to hatch within an approximate time frame of four days. Upon hatching, the newly formed larvae then burrow into the stratum corneum, where they form moulting pouches. Upon the successful moulting of the larvae into a nymph, further moulting then occurs resulting in a fully developed adult mite. The mating process begins with the male mite fertilising the adult female by penetrating through its moulting pouch. Once fertilised, the female leaves the initial pouch and resurfaces above the stratum corneum where it then begins to migrate to another suitable epidermal location. The female then begins to burrow into the epidermis where it begins to lay eggs for approximately one to two months (Fig. 5). Typically, the clinical manifestations of scabies are the primary concern for the host, however due to the pathology of this condition, social stigmas may also occur, resulting in mental and psychological issues (28). In terms of its global distribution, scabies is most abundant in tropical climates, in overcrowded environments and regions of poor healthcare.

### Tungiasis

**Fig. 6:**
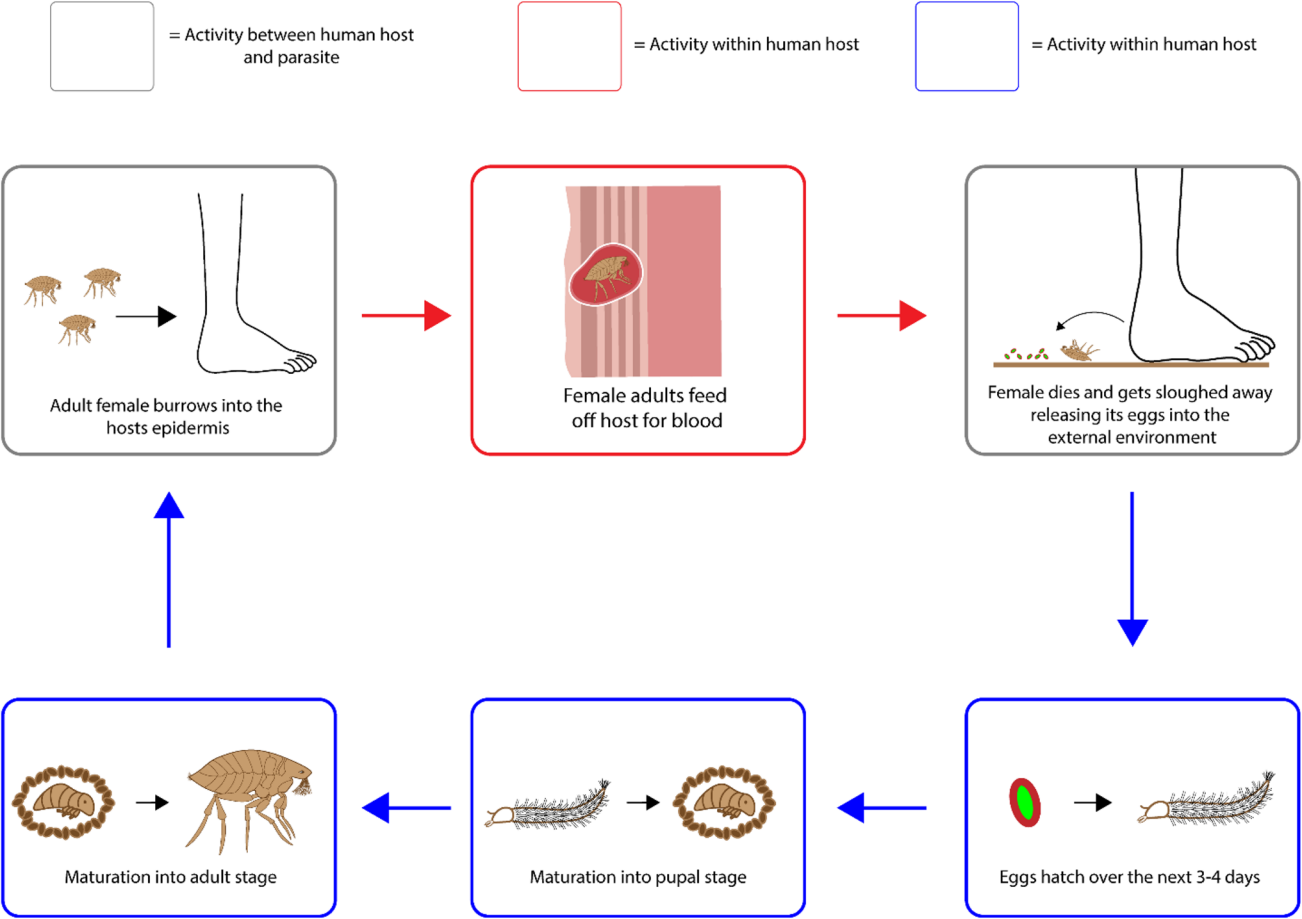
The life cycle of *Tunga penetrans* and *Tunga trimamillata*.

Tungiasis stems from the infestation of the female sand fleas *Tunga penetrans* and *Tunga trimamillata*. The life cycle begins with the sand flea penetrating through the host’s epidermal layer before burrowing into the stratum granulosum where it then begins to feed off the host’s dermal vasculature for a blood meal. As the female flea feeds, it releases approximately 200 eggs into the external environment before it dies and gets sloughed away. The eggs that have been shed by the female then begin to hatch over a period of 3 to 4 days where it feeds on the local debris before entering its maturation phase into the larval and pupal stage which takes between 3 to 4 weeks. Upon its full maturation into an adult flea, it begins to seek out a host for a warm blood meal, thus restarting the life cycle, Fig. 6. It should be noted that whilst both male and female fleas feed off the host, it is only the pregnant female fleas that burrow into the host’s skin for approximately 4–6 weeks (29). Typically speaking the wounds are focused around the lower body, with the vast majority of incidences occurring on the feet, specifically in the toes and sole of the foot (30).

The pathological complications resulting from tungiasis can be separated into primary and secondary causes, with the primary cause relating directly to the flea, whilst secondary causes relate to the aftereffects of the wound caused by the flea. The initial wounding event causes localised inflammation, leading to erythema and pruritus, with the possibility of ulceration if the flea burrows into the affected appendage. This in itself can lead the production and build of pus within the affected wound and may also lead to the deformation of the patients toes as well as the loss of toenails, with respect to the intensity of infestation. The secondary causes from the initial wounding event relates to the introduction of pathogens carried by the flea which can cause adverse effects and superinfections, depending on the bacterial strain introduced into the patient. In the case of Tungiasis, the rate of superinfection was recorded at 29% within a particular study carried out by Feldmeier et al. (31). The primary bacterial strain carried by the flea is of the *Wolbachia* genus which has an endosymbiotic relationship with the host flea. Once the flea dies, the Wolbachia is released into the wound facilitating further inflammation within the wound. The secondary bacterial strain that is sometimes known to be associated with the flea is *Clostridium Tetani*, which is responsible for the development of tetanus. This can occur when the flea comes into contact with soil infected by the clostridium bacteria, ultimately resulting in the flea acting as a carrier between the infected soil and the host. The resulting pathological complications are exacerbated in countries with inadequate sanitation and protection for the foot, leading to further opportunities for the flea and pathogens to develop. The accumulation of both parasites and pathogens can lead to the necrosis of tissue of plantar tissue, beginning with localised epidermal necrosis which can occur as a consequence of the flea’s blood meals, leading to a reduction in the transportation of oxygen and nutrients to the epidermis. The geographical distribution of tungiasis is widespread amongst sub-saharan Africa, south America, India and Pakistan where it is endemic (32).

### Gnathostomiasis

**Fig. 7:**
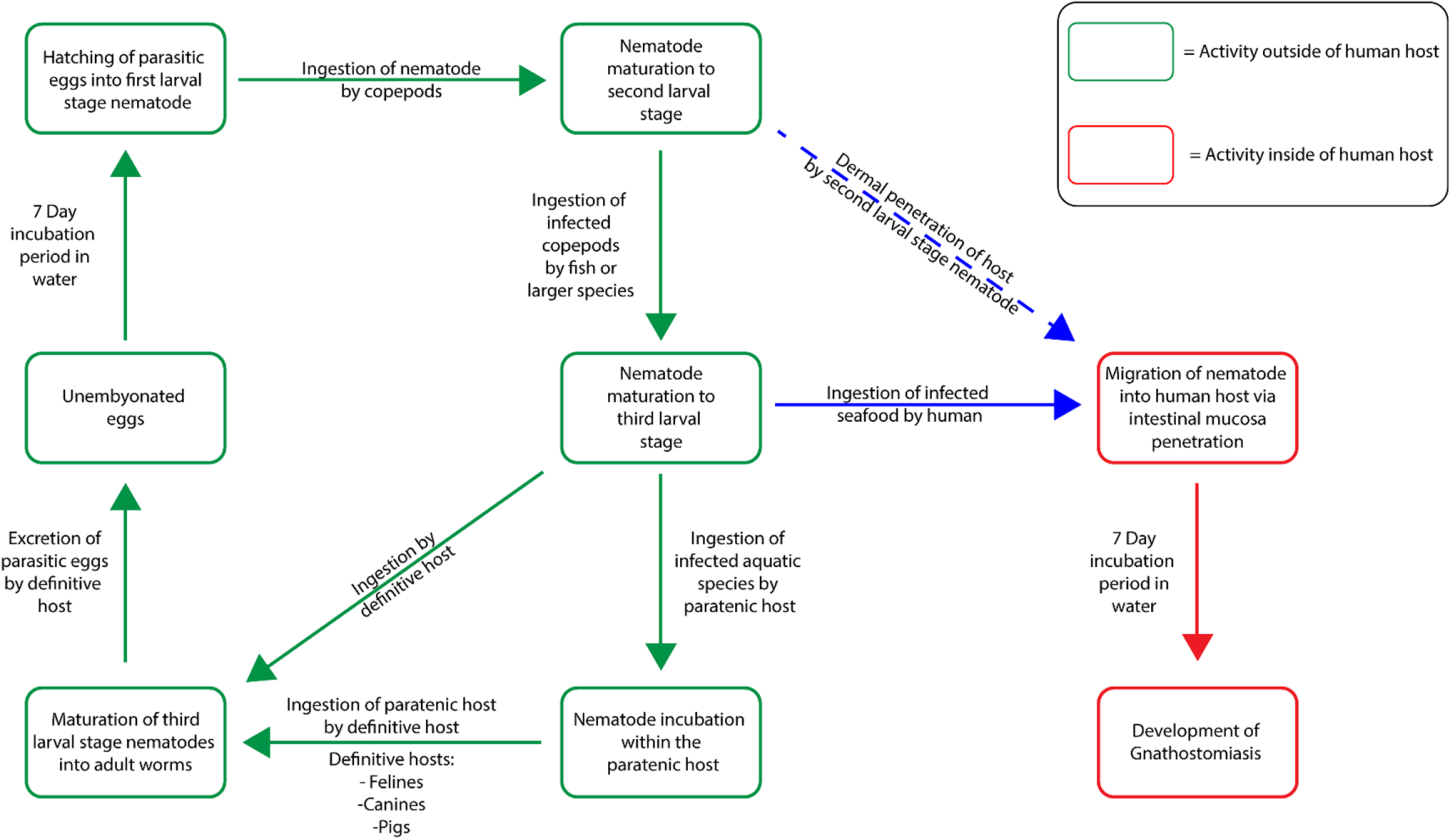
The life cycle of *Gnathostoma* nematodes.

Gnathostomiasis is the parasitic disease caused by the migratory action of third larval stage *Gnathostoma* nematodes through human tissue and are generally acquired through the consumption of raw freshwater fish and copepods. The initial cycle for the *Gnathostoma* parasite begins with the hatching of the parasitic eggs into their first larval stage which requires an incubation period of 7 days. The first larval stage nematodes are then ingested by copepods which then allow the nematodes to reach their second larval stage. This second larval stage nematode is then ingested by larger species such as fish, i.e., loach and eels, as well as reptiles and amphibians which then allow the nematode larvae to transition into the third larval stage localising within the muscle tissue of the second host (33). Humans then typically ingest the infected fish in its raw or undercooked state, which allows the third larval stage nematode to migrate from the second host into its human host via the penetration of the intestinal mucosa. This is most prevalent in certain regions of the world, where raw fish is consumed regularly as a part of the regions culture, i.e., in the form of sashimi, sushi and ceviche (34). Post-ingestion of the parasite does not illicit any immediate clinical cutaneous response until the third or fourth week, where visible signs of cutaneous injury may begin to surface as a result of the lesions caused by parasitic migration through the superficial tissue, Fig. 7. Due to the non-severe nature in which the initial cutaneous symptoms manifest, it is commonly overlooked as it is not coupled with any systemic symptoms, leading to possible cases of misdiagnosis. The initial cutaneous symptoms typically dissipate within the span of one to two weeks; however, these symptoms usually reoccur later on, manifesting near its initial site or upon the areas of the chest and abdominal region.

The migratory route of the cutaneous gnathostomiasis typically traverses the dermis and subcutaneous tissue, however in some instances the parasite itself may migrate upwards towards the epidermis resulting in the formation of a defined nodular region containing the parasite in its hibernated state (35). In this instance a punch biopsy can be undertaken to remove the nodule and thus the parasite. The defining characteristic of cutaneous gnathostomiasis is the migratory pattern of the parasite, which can be identified by visible areas of irritation, pruritus and migrating lumps (35). Generally speaking the initial site of cutaneous irritation can be anywhere on the human body, however its subsequent cutaneous manifestations will typically occur on the chest and abdominal region, with only a single migratory location undergoing cutaneous inflammation in most instances.

### Onchocerciasis

**Fig. 8:**
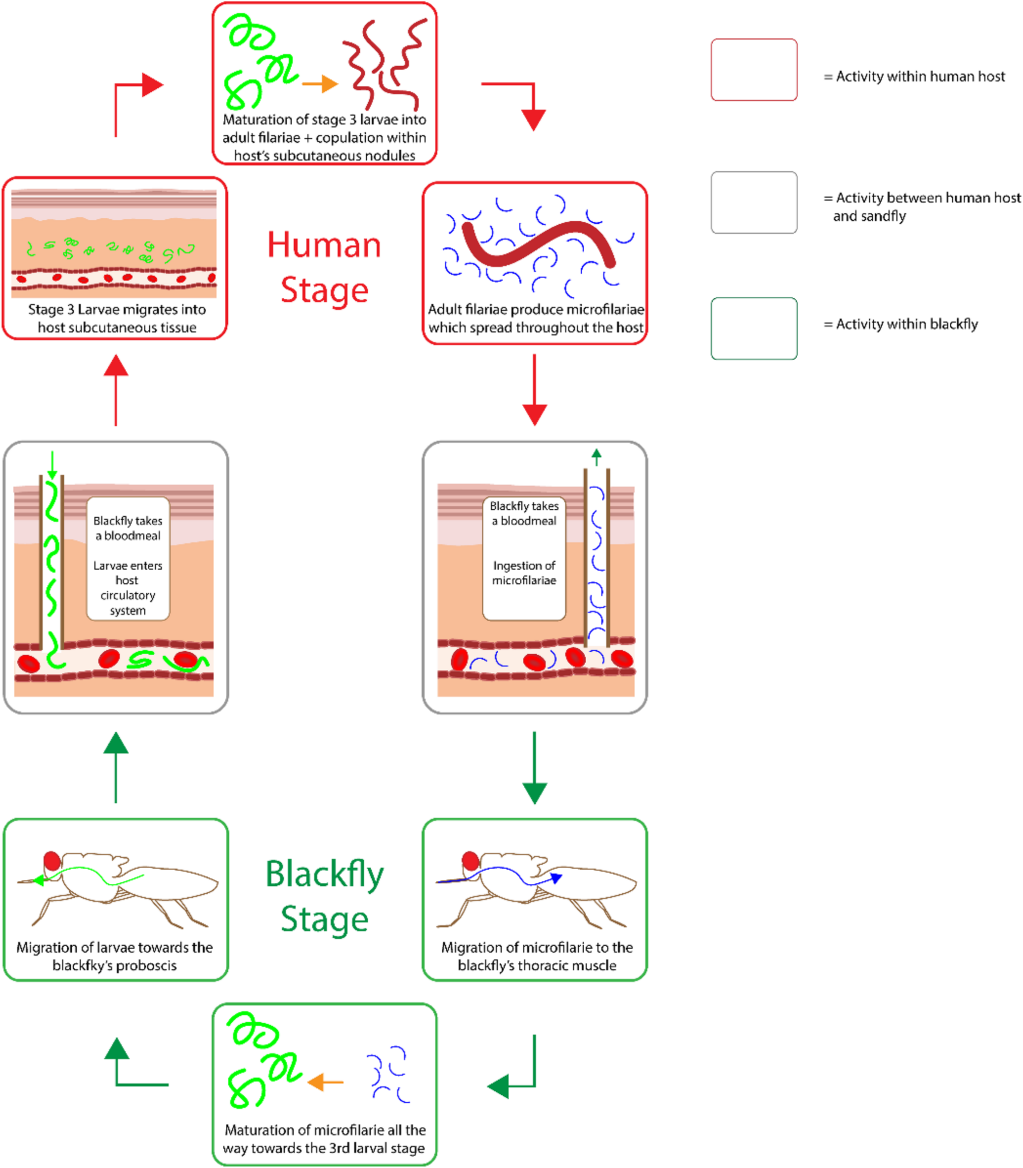
The life cycle of *Onchocerca volvulus*.

Onchocerciasis is the cutaneous parasitic disease associated with infection of the nematode parasite *Onchocerca volvulus *via a black fly of the *simulium* genus. The life cycle begins by when the female blackfly begins to feed on a human, resulting in the transmission of third stage filarial larvae from within the blackfly to the human host. From there, the larvae migrate down towards the subcutaneous tissue where they begin to mature into the adult form. These adult filariae are generally found within nodules where they can live for up to 15 years. Within that time span, female adults produce microfilariae for up to 9 years. These microfilariae are spread throughout the body, with the most common location being the skin and connective tissue but larvae have also been known to migrate towards the periphery and may be found in the blood and urine of the host. Upon the feeding of another *simulium* black fly, the microfilariae within the skin of the human host are ingested, which then migrate towards the blackfly’s midgut. From there they begin to mature into the first larval stage, all the way up to the third stage where they then enter another human host, thus repeating the cycle (Fig. 8). The main cutaneous manifestation of onchocerciasis occurs as a result of immunoreactivity towards the adult filariae and microfilariae. One of these cutaneous manifestations is the development of fibrosis around the adult filariae which induces the formation of nodules around the affected area. Cutaneous manifestations associated with microfilariae include minor tissue inflammation in the presence of live migrating parasites, whilst dead microfilariae induce more severe tissue inflammation with the possibility of necrosis (36). Overall, this results in a pruritic papular rash with hyperpigmentation and scarring on the cutaneous surface. Although the cutaneous manifestation of onchocerciasis is quite significant, the main clinical symptom is actually associated with the degradation of ocular integrity which can ultimately lead to blindness. Initial clinical presentation include a transient rash, ocular pruritus and photophobia, however if the infestation reaches a chronic level, the host may experience lichenification, tissue atrophy and the loss in vision. Lichenification is a secondary skin lesion process which can occur as a result of chronic pruritus and is characterised by the transformation of the skin into a thick leathery texture that is often accompanied by hyperpigmentation. In terms of the geographical distribution of onchocerciasis worldwide, the majority of cases occur within Africa, with some cases also found in Latin America (https://www.who.int/onchocerciasis/distribution/en/).

### Strongyloidiasis

**Fig. 9:**
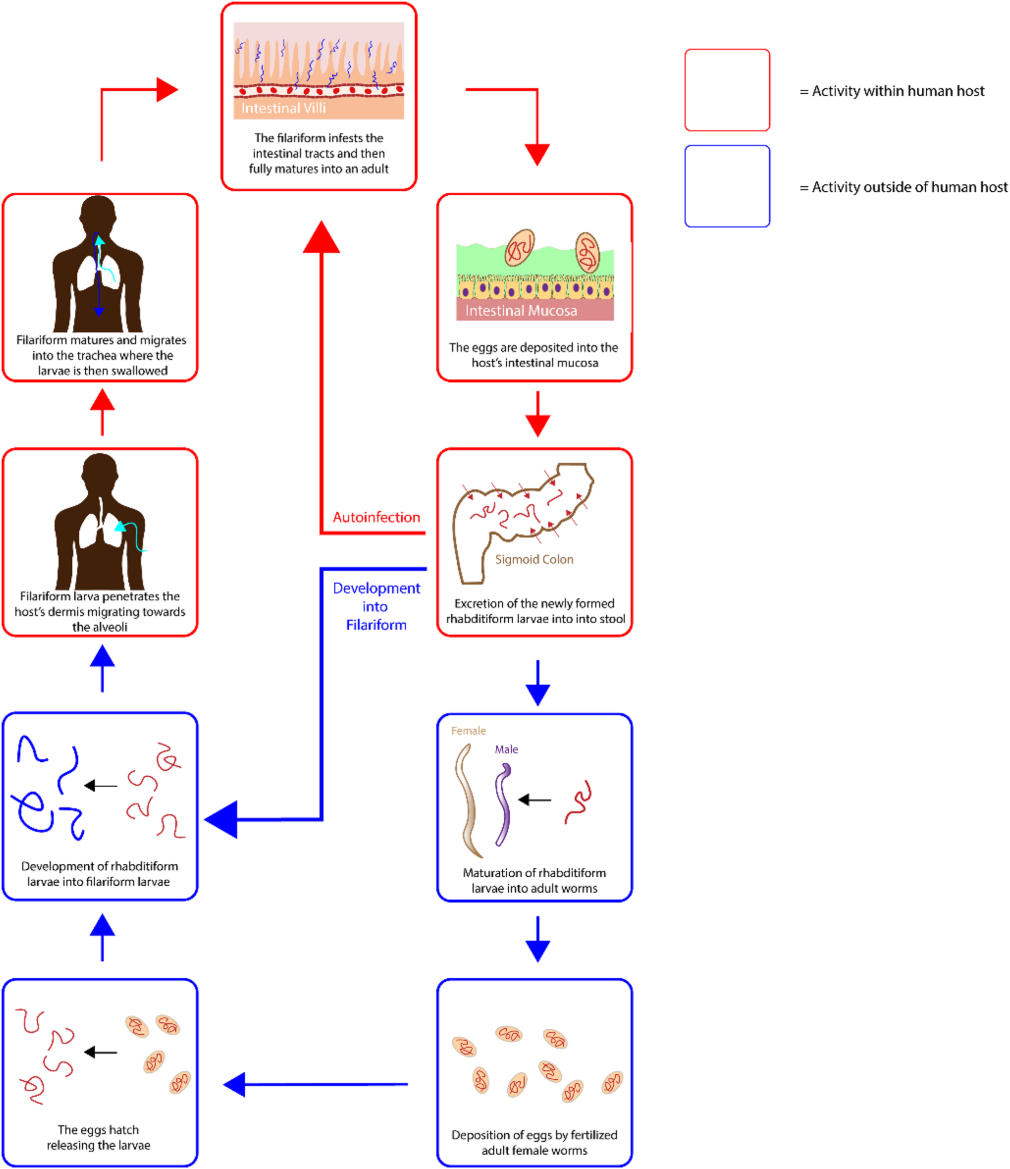
The life cycle of strongyloidiasis parasites.

Strongyloidiasis is the parasitic infection with the helminth *S.stercoralis, S.fuelleborni, S.myopotami* and *S.procyonis* which results in a variety of pathological complications. The life cycle of strongyloidiasis occurs via the infestation of the host by the filariform found within contaminated soil. This filariform larva typically penetrates through the skin of the host, migrating upwards towards the alveoli where it matures before its subsequent migration towards the trachea. Upon reaching the trachea, the host swallows the larva leading to the infestation of the small upper intestinal tract, where it matures and lays eggs within the intestinal mucosa. From here the life cycle diverges into the free-living cycle or the auto-infection cycle. In the case of the free-living cycle, the newly hatched rhabditiform larvae are non-infective and simply travel to the intestinal lumen where they are excreted, leading to soil contamination. The excreted rhabditiform larvae will then mature into filariform larvae, thus restarting the entire cycle. In the case of the auto-infection cycle, the rhabditiform larvae matures into filariform larvae within the intestinal lumen, this then results in the filariform larvae penetrating through the perianal skin, thus resulting in host reinfection, Fig. 9.

The auto-infection cycle is the most problematic as it is self-perpetuating and can therefore be extended throughout the host’s lifetime unless the appropriate treatment is facilitated. One of the most significant problems associated with strongyloidiasis auto-infection is the issue of hyperinfection syndrome. This occurs as a result of repeated auto-infection cycles which causes the host to become immunosuppressed, leading to cases of sepsis as a result of gradual bacterial infection within the damaged intestinal walls. Another issue resulting from perpetual auto-infection cycles is the positive feedback loop generated, which leads to the accumulation of filariform larvae within the body. This can ultimately lead to the dissemination of larvae within the host, resulting in the possible migration of filariform larvae towards end organs such as the brain, which can lead to host mortality. Due to the migratory nature of these parasites, other organs can be infected leading to possible cases of human-to-human transfer via organ transplants.

In terms the host’s immune reaction to strongyloidiasis, there is generally no clear symptoms, until the host reaches the hyperinfection stage or if the dissemination of filariform accumulate in other organs, thus eliciting an immune response. Hyperinfection based symptoms are dependent on the origin of infection and can be classified into gastrointestinal, pulmonary or extraintestinal. Gastrointestinal symptoms include vomiting, nausea, abdominal pain and diarrhoea, whilst pulmonary symptoms include haemoptysis, tracheal irritation, coughing, dyspnea and wheezing. Extraintestinal symptoms can also be subdivided into skin, central nervous system, haematological and allergic response. The main cutaneous symptoms are pruritus and petechial rashes, central nervous system symptoms include seizures, headaches and comas, haematological symptoms include chills and fevers, whilst allergic responses can result in hives or anaphylaxis (37). In rare cases strongyloidiasis can elicit an acute symptomatic response upon the immediate exposure to the parasite, whereby the symptoms can prolong for up to several weeks. The geographical distribution of strongyloidiasis is most prevalent within the regions of southeast Asian, sub-Saharan Africa, southern and eastern Europe, the Caribbean islands and Latin America. Overall, there has been a global increase in strongyloidiasis due to a variety of reasons ranging from the lack of sanitation, insufficient supply of potable water, poor hygiene etc.

## Comparisons between cutaneous parasites

The general consensus regarding the similarities and differences between cutaneous parasites relate to their specific associations with the host. Each parasitic scenario is distinguishable from one another based on their cutaneous symptoms, duration, possibility of reinfestation, as shown in Table II. Generally speaking the majority of parasites that elicit significant cutaneous symptoms enter the host through trans-epidermal means, triggering a minute immune response in most cases. However, this initial response is generally a result of either the vector or microparasite piercing into/through the epidermis, so the actual parasite associated pathological symptoms do not take effect until later on. The dormancy period is specific to the parasite in question whereby some cutaneous manifestations do not appear until several months have elapsed. The nature of these dormancy periods are dependent on the lifecycles of the parasite and their associated behaviours in regards to its interactions with the host. For example, the infestation of a microparasite, such as the strongyloidiasis parasite rarely elicits any immediate major cutaneous responses and lays dormant for extended periods of time before the eventual mass accumulation of filariform, which triggers the discernible cutaneous symptoms. On the contrary another microparasite such as the one responsible for onchocerciasis, can require a couple of days before the immune system detects any significant activity relative to the adult filariae and microfilariae, which then leads to the manifestation of cutaneous symptoms. On the other side of the spectrum, there are macroparasites such as the one responsible for tungiasis which typically elicits an immediate immune response due to the magnitude of epidermal damage caused by the infestation process. The classification criteria of microparasites and macroparasites does not simply depends on the size of the parasite itself, but also on its lifecycle with respect to the location of its reproductive cycle. Macroparasites can typically be distinguished by the fact that they reproduce outside of the host, whereas microparasites almost always reproduce from within the host. Based on the information displayed in Table II, the parasites are quite distinguishable from one another based on the time period associated with the manifestation of cutaneous symptoms, as well as their specific pathological symptoms.

**Table II:** Similarities and differences between cutaneous parasites

	Gnathostomiasis	Leishmaniasis	Lyme disease	Myiasis	Onchocerciasis	Pediculosis	Scabies	Strongyloidiasis	Tungiasis
Parasite enters through the skin	N	Y	Y	Y	Y	Y	Y	Y	Y
Immediate dermal reaction at the point of infestation	N	Y	Y	Y	N	Y	N	Rare case	Y
Delayed dermal reaction post infestation (Days-weeks)	Y	N	Y	N	Y	N	N	N	N
Significantly delayed dermal reaction post infestation (Months-years)	Y	Y	N	N	Y	N	Y	Y	N
Continual cycle of reinfestation	N	N	N	N	N	Y	Y	Y	N
Parasite intentionally exits the host	N	N	N	Y	N	Y	Y	N	N
Retainment period of parasite within the host	Up to 12 months	Up to 3 years	36–48 h	5–10 weeks	Up to 15 years	Up to 30 days	2–3 months	Perpetual	4–6 weeks
Significant cutaneous migration?	Y	N	N	Y	Y	N	N	Y	N
Significant cutaneous damage occurs as a direct result of parasitic migration through hosts skin	N	N	N	Y	N	N	N	N	N
All damage is localised at initial wound site	N/A	N	N	N	N	Y	Y	N	Y
Specific cutaneous symptoms	Meander like lesions	Nodules, ulcers and plaques	Large concentric erythema	Meander like lesions or furuncles	Nodules, fibrosis, lichenification, hyperpigmentation	Crusted papules, maculae ceruleae	pruritus, crust formation	pruritis and petechial rashes	Inflammation, ulceration

## Current treatments

The general route for treatment of cutaneous parasites typically involves the use of antibiotics to combat parasite associated pathogenesis. In most cases, the oral route of drug delivery is most common for combatting cutaneous parasites, due to the systemic nature of parasitic circulation and distribution within the host. Whilst oral administration is the most common drug delivery pathway, other delivery methods can also be used for effective administration. Intravenous delivery is utilised for certain cutaneous parasites such as *leishmaniasis*, Lyme’s disease and onchocerciasis, whilst intralesional drug delivery is applied specifically to *leishmaniasis.* Other methods include topical antibiotic delivery as well as more miscellaneous methods such as suffocation heat therapy and larvae removal.

### Antibiotics

Anti-parasitic strategies via the use of antibiotics is the most common method, given the fact that antibiotics are systemically distributed which ensures that the parasite will be affected within a certain time period before the antibiotic is excreted from the host. Currently, most of the utilised antibiotics focus on the disengagement of the parasite from its usual functions such as procreation and migratory movement. In this regard most antibiotics do not actively kill the parasite, but instead reduces it such a state whereby it can no longer reproduce or undertake the necessary actions to survive. The most commonly used antibiotics are outlined below.

Ivermectin is a synthetic anthelmintic drug with a broad spectrum of antiparasitic activity. Ivermectin works by selectively binding to chloride ion channels within the nerve and muscle cells of microfilaria which in turn increases the permittivity of the microfilaria cells towards chloride ions, thus resulting in a cellular hyperpolarisation and therefore cell death. Ivermectin is most commonly used for gnathostomiasis, myiasis, onchocerciasis, pediculosis, scabies and strongyloidiasis. In terms of its regimen, varying oral dosages are used for different parasites. For gnathostomiasis 0.2 mg/kg for 7 days, myiasis on to two doses of 150–200 mg/kg, onchocerciasis 150 mg/kg every 6 months, pediculosis 200 mg/kg every 10 days, scabies two doses of 200 µg/kg at an interval of two weeks and strongyloidiasis 200 mg/kg, daily for 2 days (2, 38–42).

Albendazole is anthelmintic drug that has multiple mechanisms for the induction of anti-parasitic activity. Albendazole selectively degenerates the cytoplasmic microtubules via the inhibition of microtubule polymerisation which prevents the parasitic cells from undergoing mitosis, ultimately killing them. The other mechanisms include the disruption of metabolic pathways which inhibits ATP synthesis, as well as the disruption to the parasites glycogen storage which prevents the parasite from effectively utilising glucose. One of the issues regarding albendazole lies in the fact that it has a very low solubility within water, so for the oral administration route, it is generally suggested for albendazole to be ingested alongside meals with high fat content. Current usages include gnathostomiasis 400 mg/kg for 21 days, myiasis 400 mg/k for 3 days, strongyloidiasis 400 mg twice daily for 7 days (2, 38, 42).

Fluconazole and ketoconazole are both orally administered antifungal drugs used in the treatment of systemic and cutaneous fungal infections. Their mechanism of action are exactly the same, where both pathways follow the selective inhibition of the enzyme lanosterol 14-α-demethylase which is used for the conversion of lanosterol to ergosterol. The inhibition of this enzyme ultimately prevents the formation of the fungal cell wall which requires the use of ergosterol in its synthesis. In the specific case of their usage against cutaneous parasites, its primary usage is in the improved healing of cutaneous lesions via the suppression of fungal growth. For its application towards cutaneous *leishmaniasis*, 200 mg of fluconazole should be consumed daily for a duration 6 weeks, whilst ketoconazole should be consumed 600 mg daily for 28 days. Unlike fluconazole, ketoconazole has noticeable gastrointestinal side effects, leading to its eventual replacement by fluconazole (43).

Miltefosine is an antimicrobial agent that is specifically utilised for *leishmaniasis*. The mechanism of action follows the disruption of normal mitochondrial function through the inhibition of cytochrome c oxidase which results in cell death. For the treatment of *leishmaniasis*, 50 mg of miltefosine should be daily for 28 days, however it should be noted that certain gastrointestinal side effects such as nausea and vomiting may occur (43).

Amphotericin B is an antifungal drug that can produce fungicidal or fungistatic effects depending on the concentration of the dose relative to the susceptibility of the fungal target. Unlike fluconazole and ketoconazole which targets the production ergosterol, amphotericin B specifically targets the ergosterol itself by to it, thus destabilising the integrity of the cell membrane which leads to the formation of transmembrane channels which in itself causes the contents of the fungus to leak out, resulting in cell death. Amphotericin B is used in the treatment of *leishmania* through intravenous injection of 0.5–1.0 mg/kg per day for 20 days (43).

Sodium stibogluconate is an anti-*leishmania* drug that can be applied through the intravenous and intralesional pathways. The mechanism of action follows the inhibition of DNA topoisomerase which is vital for DNA replication and transcription. This is because DNA topoisomerase controls the release and recombination of the DNA strand, which if inhibited prevents the cell from replicating leading to cell death. Sodium stibogluconate should be applied intravenously 20 mg/kg for 20 days, whilst intralesional application is 0.2–5 ml every 3–7 days (43).

Paromomycin is an antibiotic that inhibits bacterial protein synthesis. The mechanism of action follows the binding of paromomycin to the 16 s ribosomal RNA which then results in the formation of defective polypeptide chains during the protein synthesis. This eventually leads to the build of defective proteins within the bacterial system, thus resulting in bacterial death. For its usage against *leishmania*, paromomycin should be applied topically at a concentration of 15% coupled with + 12% methylbenzethonium chlorideointment for a duration of 10 days, then 10 days off, then finally 10 days application (43).

Doxycycline is a synthetically derived antibiotic used in the treatment of a wide range of bacterial infections. The mechanism of action follows the binding of doxycycline onto the 16 s rRNA area of the bacterial ribosome which is responsible for protein synthesis. Once bound, the 16srRNA portion is unable to bind to RNA-30 s which ultimately prevents protein translation from occurring. Overtime this prevents the bacteria from replicating, thereby producing a bacteriostatic effect. For its application against Lyme’s disease, doxycycline is to be taken orally 100 mg twice daily for two weeks, whilst the intravenous injection of doxycycline should only be used in severe cases (44).

Amoxicillin is an antibiotic derived from penicillin for the treatment of gram-positive bacteria. Amoxicillin works by inhibiting the continual cross-linkage of the bacterial cell wall through the disruption of penicillin binding proteins. Overtime the bacterial cell wall weakens due to the imbalance between enzyme based autolytic action and cross-link maintenance, ultimately leading to the leakage of the bacterial organelles and thus cell death. For its usage against Lyme’s disease amoxicillin 500 mg is taken 3 times per day for 2 weeks, whilst intravenous injections are used in the most severe cases (44).

Cefuroxime is a beta-lactam antibiotic that covers a broad spectrum of bacterial infections, similar to that of penicillin. The antibacterial mechanism of cefuroxime follows the inhibition of the bacterial wall synthesis process, specifically that of the third and the final stage. This disrupts the formation of peptidoglycan layer that makes up the bacterial cell wall which leads to bacterial cell death through the leakage of its internal content. For Lyme’s disease, Cefuroxime is prescribed 500 mg twice per day for 2 weeks, whilst intravenous injection is used for severe scenarios (44).

Mebendazole is an anthelmintic used to treat the infection from parasitic worms such as myiasis (44). The mechanism of action works by directly preventing the parasitic worms from producing microtubules which are needed to facilitate the absorption of glucose when the worm is in its larval and adult stages. Mebendazole binds to tubulin preventing it from undergoing polymerisation which in itself prevents the formation of microtubules. As a result of the parasite being unable to uptake glucose it eventually depletes it energy storage and dies as a result.

Levamisole is an anthelmintic drug designed to treat bacterial and viral infections from parasitic sources such as myiasis (44). Levamisole specifically targets the nicotine receptors as a way of facilitating its mechanism of action against parasites. The specific action that is facilitated by levamisole follows the severe reduction in copulative capacity, via the inhibition of the male parasite from using its reproductive muscles, thereby preventing copulation from occurring. Other benefits also include the stimulation of host-cell activation, coupled with improved phagocytotic functions, however it has been withdrawn from the market due to a variety of adverse effects.

Moxidectin is a semisynthetic antiparasitic drug that works against both endo and ectoparasites. The mechanism of action works via the specific binding of the chloride ion channels within the parasite which are required for the normal functioning of nerve and muscle cells. After moxidectin had been bound to the parasite, the ion channels become more permeable resulting in a high increase of chloride ions within the parasite, leading to its paralysis and its eventual demise. Moxidectin is generally prescribed in 8 mg doses with varying dosage periods depending on the severity of onchocerciasis, however it has been replaced by ivermectin in most cases (39).

Despite the efficacy of antibiotics, they can also produce significant side effects which occur as a result of their systemic distribution within the host. This issue coupled with the development of antibiotic resistance result in a situation whereby antibiotics can no longer be considered as a sustainable anti-parasitic method, which further incentivises for the development of a localised anti-parasitic treatment that specifically targets the parasites instead of resulting in unwanted systemic effects. Therefore, there is an urgent need to develop alternative cost-effective treatment methods for patients with cutaneous parasite infection.

### Advanced therapies

#### Thermotherapy

Thermotherapy works on the basis that some parasitic species such as the *Leishmania* species are unable to multiple within the host’s macrophages once the localised temperature is above 39 °C (45). The typical approaches to this method include the utilisation of infrared light, hot baths and laser therapy, all of which can generate non-localized heat that can damage the other tissue surrounding the cutaneous lesion of interest (46). Due to such reasons, radiofrequency-based thermotherapies were developed as a means of more accurately targeting *leishmanial* lesions without affecting surrounding tissue, thus leading to higher quality treatment with minimal side effects. Generally speaking radio frequency based thermotherapy is the most effective, where some studies have shown that a single application was able to encourage the reepithelization of the *leishmania* affected lesion, thus improving the speed of healing (47). It should also be noted that the utilisation of radio frequencies can also help to stimulate increased collagen synthesis, contraction and remodelling which ultimately results in improved cutaneous healing with significantly better cosmetic results (48). Despite the various advantages presented by radio frequency induced thermotherapy, the main limitations follow the fact that radio frequencies only penetrate to a depth of 4 mm which is ideal for *leishmania* amastigotes, but not for other cases of *leishmaniasis* that have penetrated deeper into the subcutaneous tissue, thereby limiting its usage to only superficial *leishmaniasis*. Although the main reported applications of thermotherapy are in the treatment of cutaneous leishmaniasis, this may also be used to kill other parasite infections such as *gnathostomiasis*, *myiasis*, and *sarcoptes scabiei,* where literature on the use of heat in cooking fish (https://web.stanford.edu/group/parasites/ParaSites2001/gnathostomiasis/PAGES/index.html) or ironing clothes (49) or direct heating (https://www.cdc.gov/parasites/scabies/gen_info/faqs.html) are used as a thermal mechanism to kill parasites in fish have been reported.

#### Cryotherapy

Cryotherapy is an alternative treatment for *leishmaniasis*, which typically either utilises liquid carbon dioxide or liquid nitrogen to kill the parasites. The suggested mechanism of action works by freezing the parasite, which causes the formation of intra and extracellular ice crystals, which when fully expanded rupture the parasite’s cell membrane (50). This has shown to be quite effective in regard to the facilitation of amastigote based cryonecrosis. Despite the fact that this form of treatment has been reported to have a success rate of above 95%, controlled trials have instead shown its effectiveness to be approximately around 27% (51, 52). This low clinical success rate may be attributed to a variety of different factors, such as the fact that this cryotherapy does not immediately contact the dermis due to the Leidenfrost effect, which in turn reduces the efficacy of the therapy, as immediate contact is required to eliminate the parasites without damaging the surrounding cutaneous tissue (53). Other factors include the duration and frequency of each cryotherapy session, as the duration of each liquid nitrogen application may be too short between each interval to effectively inhibit the proliferation of parasites within the affected lesions (52). Similar effects are observed when cryo-treating patients *with tunga penetrans* (54). Although efficacy is observed against *leishmaniasis and tunga penetrans*, cryotherapy is not recommended for other cutaneous parasites such as *gnathostomiasis.*

#### Photodynamic Therapy

Photodynamic therapy in the context of cutaneous anti-*leishmanial* treatment refers to the utilization of photo-excitable dyes in conjunction with specific wavelength frequencies to induce the release of reactive oxygen species (ROS), which in turns results in the photodynamic inactivation of the parasites. In the case of *Leishmania*, the dyes uroporphyrin and phthalocyanines are utilised to facilitate complete deactivation (55). Other dyes include methylene blue which can serve as a low-cost alternative for photodynamic therapy (56). The main advantage of this therapy is that the dye can selectively accumulate within the parasite prior to the application of the ROS inducing wavelengths. This allows for the effective destruction of parasites without harming the host tissue. Photodynamic therapy is also being used in the quest to eradicate Lyme’s disease (https://www.klinik-st-georg.de/en/antimicrobial-photodynamic-therapy/).

#### Laser therapy

The application of lasers for anti-parasitic treatment is dependent on the type of laser that is used, which determines its output, oscillation form and conversion efficiency. Output refers to the strength of the laser in megawatts, whilst oscillation form refers to the motion of the laser which can either be pulsed of continuous. Conversion efficiency refers to balance in the energy input with respect to useful energy output. Lasers are classed into 3 different types, gas lasers, solid-state laser and semiconductor lasers, however only gas and solid-state lasers are used for antiparasitic treatments. Gas lasers utilise gas as its laser medium, which in the specific context of anti-leishmanial treatment, either requires the use of carbon dioxide or argon. Solid-state lasers use ores as the laser medium, which in the context of anti-leishmanial treatment uses neodymium-doped yttrium aluminium garnet (Nd:YAG) or erbium (57).

The general consensus is that carbon dioxide is the most commonly utilised compound for *leishmanial* laser therapy, which is primarily due to its abundance in nature, coupled with its effectiveness and safety when used for *leishmania*. The only problems associated with carbon dioxide lasers, are the minor side effects which are generally of a cosmetic nature, such as hypertrophic scarring, erythema and hyperpigmentation (58). The other compounds also elicit similar results to carbon dioxide, but with differing levels of efficacy. An advantage of using laser therapy lies in the fact that the power density can be varied to induce various effects upon the affected lesion i.e., the affected lesion can be ablated to destroy the parasites and can then be treated again with a lower power density laser to form a protective top layer which will isolate the freshly ablated tissue from the external environment. Another form of laser therapy is the use of pulsed dye laser which improves the cutaneous properties of the lesion, resulting in improved pliability, reduced lesion size, reduction in erythema and improved skin texture, however one of the problems of pulsed laser dyes lies in its limited penetration depth, which constrains it to only superficial applications (59). The limitations associated with laser penetration depth is primarily dependent on the laser medium and the wavelength that is used, as opposed to the oscillation form. An example would be Nd:YAG at differing wavelengths, where by 2940 nm only results in partial penetration through the stratum corneum, whilst at 1064 nm results in the laser penetrating through to the dermal vasculature layer (60).

#### Nanoparticle based therapy

There is large scope within nanotechnology to help in the development of new platforms. These may be either based on drug carriers such as liposomes, polymeric micelles or dendrimers, incorporated into larger macromolecular structures such as into hydrogels, wafers or even into bandages. The standard delivery method of anti-parasitic compounds typically follows the ingestion route which leads to the systemic circulation of the compound, resulting in lower efficacy, non-specificity and increased side effects. To combat this issue, nanoparticles have been utilised as a means of increasing the efficacy of drug delivery whilst reducing the levels toxicity. In the case of *leishmaniasis* treatments, it has been reported that a variety of different nanoparticles have shown significant effectiveness against the parasite. Such nanoparticles include liposomes, metal oxides and lipid nanoparticles, with iron (III) oxide magnetic nanoparticles being of particular interest due to their ease of removal from the host’s body thereby making them a good carrier medium for superficial drug delivery (61, 62). For drug delivery, liposomal preparations of antimicrobials such as amphotericin B have been reported for *leishmainia* treatments (63, 64), whilst polymeric carriers have been reported loaded with primaquine (65) or amphotericin B (66). Combined delivery of antibiotics using nanotechnology delivery systems has resulted in reduced resistance (67), these findings can be used to guide the development of interventions of new anti-parasitics. Whilst there is some progress in this field there is huge scope to improve and widen the target from *leishmaniasis* to other parasites.

Aside from drug delivery, nanotechnology can be used topically as a local lethal dose killing off parasite activity. Iron (III) oxide nanoparticles have displayed anti-*leishmanial* effects, in which the suggested mechanism of action occurs through the production of nitric oxide (68). Nitric oxide is one of the main molecules utilised by macrophage against *leishmania*, which involves the macrophage undertaking the oxidative burst mechanism. This produces high quantities of nitric oxide and ROS which effectively facilitates the elimination of promastigotes within the macrophage, thereby limiting the population size within the host (69). Whilst the ROS induced mechanism of anti-parasitic activity is well understood, the same does not apply to nitric oxide as its specific mechanism is still not fully understood. Current research indicates that nitric oxide is not directly involved in the direct killing of *leishmaniasis* and may instead contribute to host tissue damage (70), however it has also been shown that downregulation of nitric oxide provides *Leishmania* with a form of immune escape via reduced host response (71), thereby suggesting that nitric acid is needed to prevent immune escape, thus implying that nitric acid is needed to initiate a host response against the parasite. Other studies have examined the use of silver nanoparticles exploiting their inherent antimicrobial / localised cytotoxicity either alone or in combination with UV light (72, 73).

Another exciting application of nanotechnology for treatment of cutaneous leishmaniasis is the use of iron oxide coupled with magnetic flux, this results in magnetic hyperthermia which can be used to kill parasites. Berry et al*.* reported the use of iron oxide as heat seeds for thermal kill of amastigote cells *in vitro* (74). This study in combination with the ROS generation finding, indicates that iron oxide nanoparticles may be a frontrunner in the next generation of *leishmania* treatments.

The literature in this area is highly biased towards leishmania treatment, however, there is scope to develop therapeutics for other cutaneous parasites. The beauty of nanotechnology lies within the breadth of unique qualities each material possesses at the nano-scale domain, as well as the ease of tailor-ability towards bespoke applications. We believe that more work targeted in this area towards some of the less studied parasites may render great reward.

## Future Perspectives

The current treatments for cutaneous parasites are virtually all encompassed by the use of drugs as a general solution. Alternative treatments are effective, but nonetheless are limited to a specific type of parasite. The primary issue that is inhibiting the development of a general purpose non-antibiotic based therapy lies in the fact that all cutaneous parasites have different life cycle mechanisms, coupled with varying migratory routes which may not result in the parasites having an extended period of time whereby they dwell within the superficial layers of skin. For a parasite such as *leishmaniasis*, which dwells within the superficial layer of skin, non-invasive treatments can be applied to a high level of efficacy as the parasite lives within the cutaneous nodules, thereby acting as a viable point for exploitation. Other parasites have shown to pass through the upper cutaneous region during their migratory routes, however unlike *leishmaniasis* and possibly onchocerciasis, other parasites are not known to live within exposed regions of the host and as of such cannot be treated through alternative treatments. Perhaps a consideration that needs to be taken is our current methods for approaching parasitic treatment. Our current alternative methods aim to target the parasites based on where they reside, which presents us with a specific set of limitations, specifically the depth and invasiveness of the treatment that can be applied. Instead, it may be worth considering developing a method which instead influences the migratory route of the parasite, thereby herding them to a specific area where they can then be annihilated in a more efficient manner. Whilst this method itself may not specifically partake in the direct destruction of the parasite; it will instead act as a process to facilitate the controlled movement of parasites, which will hamper their development as a bare minimum. The primary concern regarding this method would be the use of an effective antiparasitic agent that does not compromise the safety of the host. This compound would be required to fulfil two specific requirements, one of which is for it to be non-cytotoxic to human cells and the second is for the compound itself to be able to be systemically circulated around the host before it is safely excreted out. The compound itself should also be able to exude a repulsive effect towards the parasite, which would therefore allow the migratory route to be influenced. Assuming that the compounds will temporarily accumulate in certain regions of the host, it will therefore act as a temporary road-block within the parasites migratory route, forcing them to undertaken a different migratory path. The main issue of this method is that there are currently no clinically known compounds that would have such effects and would also require a significant amount of time and resources to identify the changes in migratory pattern. Despite the significant problems associated with this method, it may be feasible with the use of magnetic nanoparticles, whereby their distribution within the host can be controlled through the use of an attunable magnetic field.

Ultimately, there is an urgent need for new pragmatic treatment approaches to parasitic infection. Often such cases present in tropical climates or low-income countries, both of which may result in challenges for administration. Biomaterials research and expertise has vastly grown over the past two decades, with solution based approaches to multiple clinical conditions or disease states. These platform technologies could be adapted to suit the requirements for the treatment of cutaneous parasite infections, however, greater awareness of the clinical need is required in order to leverage greater research investment for such progress to be realised.
